# Metabolic Syndrome and Cardiometabolic Risk Factors in the Mixed Hypercholesterolemic Populations with Respect to Gender, Age, and Obesity in Asir, Saudi Arabia

**DOI:** 10.3390/ijerph192214985

**Published:** 2022-11-14

**Authors:** Ahmed Ezzat Ahmed, Awad Alsamghan, Maha Abdullah Momenah, Haifa Ali Alqhtani, Nouf Arkan Aldawood, Mohammed A. Alshehri, Abdulaziz Mohammad Ali Alshehri, Sadeq K. Alhag, Yasser O. Mosaad, Hassan Ahmed

**Affiliations:** 1 Department of Biology, College of Science, King Khalid University, Abha 61413, Saudi Arabia; 2 Department of Theriogenology, Faculty of Veterinary Medicine, South Valley University, Qena 53823, Egypt; 3 Family and Community Medicine Department, College of Medicine, King Khalid University, Abha 61413, Saudi Arabia; 4 Department of Biology, College of Science, Princess Nourah bint Abdulrahman University, P.O. Box 84428, Riyadh 11671, Saudi Arabia; 5 Biology Department, College of Science and Arts, King Khalid University, Muhayl Asser, Abha 62529, Saudi Arabia; 6 Department of Pharmacy Practice and Clinical Pharmacy, Faculty Pharmacy, Future University in Egypt, Cairo 11835, Egypt; 7 Department of Physiology, Faculty of Veterinary Medicine, South Valley University, Qena 53823, Egypt

**Keywords:** obesity, dyslipidemia, diabetes, T2DM, hypothyroidism, HTN, CVD, TyG

## Abstract

This record study aimed to investigate the prevalence of metabolic syndrome (MetS) profiles regarding sex, age, and obesity for the riskier factor of cardiovascular diseases in a general population in Saudi Arabia. Laboratory and anthropometric measurements were performed on non-specific participants with variant ages and BMI in either sex. Serobiochemical changes were measured for metabolic profiles, i.e., A1C/FSG, TC, TGC, HDLC/LDLC, Vit.D, TSH/T4, Hb, and Cr. The study was applied in a Polyclinic, Abha, Saudi Arabia in 2020 G. The general population showed variable incidences of MetS profiles, such as 69.4% diabetes, 85.5% hypothyroidism, and 92.2% obesity. Hypothyroidism showed a higher incidence in women rather than in men, but men were more dyslipidemic, with higher TGC and LDLC but low HDLC, compared to women. Men <40 Y. showed diabetes and hypothyroidism, but elders were dyslipidemic. Women <40 Y. showed anemia and hypovitaminosis-D but were suffering from hypothyroidism at all ages. Diabetes, hypothyroidism, hypovitaminosis-D, and dyslipidemia were the main MetS components in both overweight and obese participants, and an incidence of more than 50% in each profile was recorded. Diabetes with hypertension was characteristic of obese participants rather than those overweight. About 66.1% of the mixed-hypercholesterolemic cases were diabetic, but 18.9% of the mixed-diabetic participants were hypercholesterolemic. Castelli’s risk factors, CRI-I and CRI-II, and atherogenic indices, AIP and AC, were measured for evaluating the cardiac risk in different populations based on the AUC–ROC and cut-off values. Insulin-resistance marker (TyG) was also measured, showing considerable cut-off values for diabetic susceptibility in the lipidemic participants with higher TGC and TC rather than HDLC or LDLC. In conclusion, MetS showed higher susceptibility to sex and age with increased incidence in women rather than men. However, the cardiac risk was more susceptible to men of higher TGC and low HDLC than women. Type 2 Diabetes mellitus (T2DM) was more prominent in both elders (≥40 Y.) than younger ages of either sex. Anemia and deficiency of Vit. D was characteristic of young women (<40 Y.). Hypothyroidism affects young men <40 Y. but was recorded in women of all ages. Both dyslipidemia and diabetes could trigger CVD, showing higher cardiac risk in mixed-hypercholesterolemic men rather than women. Our study strongly suggests that the consumption of unhealthy junk food, tobacco smoking, lack of exercise, and physical inactivity could be conclusive evidence of MetS in the Saudi population.

## 1. Introduction

Metabolic syndrome (MetS) is a consensus of insulin metabolic disorder, overweight, obesity, dyslipidemia, and hypertension. MetS demonstrates three major components of dyslipidemia, i.e., increased triglyceride-rich lipoproteins, decreased high-density lipoprotein (HDL), and increased low-density lipoprotein (LDL) particles [1]. It gives rise to the development of various cardiovascular diseases (CVD) such as cardiac arrhythmias, heart failure, atherosclerosis, and thrombosis [2]. MetS is characterized by insulin resistance; type 2 diabetes (T2DM), associated with obesity, is the main contributor to the syndrome at variant ages, especially in elder people [3]. In 2006, the International Diabetes Federation (IDF) recorded that up to 25% of the global population had MetS, with insulin resistance as an important risk factor for the syndrome [4]. Thus, T2DM, obesity, and hypertension were known as the major components of MetS predisposing to CVDs. 

Prevalence of MetS in different populations and ethnicities is periodically reported by international health organizations, i.e., Mexican Americans (31.9%), Caucasians (23.8%), African Americans (21.6%), and other races (20.3%) [5]. According to a previous report by National Cholesterol Education Program (NCEP), about one third of middle-aged men and women in the USA were suffering from MetS [6]. Moreover, according to the National Cholesterol Education Program–Adult Treatment Panel III (NCEP–ATP III) and IDF criteria, Gulf countries showed a progressive increment in MetS prevalence, i.e., 17% in Oman [7] and up to 40.5% in the Emirates [8]. However, in Saudi Arabia, Al-Nozha et al. [9] reported that MetS was recorded at 39.3% in 2005, depending on the criteria previously involved in the 2001 report of ATP III. A recent record study by Al-Rubeaan et al. [10] showed an increased MetS prevalence in Saudi Arabia at 39.8%, with 29.2% in women and 34.4% in men. However, that record decreased to 31.6%; 35.4% in women and 45.0% in men, according to IDF. In previous reports, around 20–25% of the adult population in the world have MetS, which increases the mortality rate among those patients that are twice as likely at risk from a heart attack and three times as likely from a stroke, rather than people without MetS [11]. 

However, the presence of MetS alone could predict 25% of all new-onset CVD [12] with variable cut-off values of MetS’ metabolic components [13]. Although MetS has become widely distributed in parallel to sedentary lifestyles and overweightness worldwide, it needs more investigation [14]. There is clear evidence that insulin resistance and obesity are the main etiologic factors of MetS with an interactive predisposition of genetics and other environmental factors [15]. The WHO reported that higher CVDs mortalities were recorded among 35- to 70-year-old people with a history of MetS and T2DM [16]. Both CVDs and diabetes involved in MetS require more investigation regarding other relevant factors affecting public health in different ages and gender [17]. MetS developing coronary heart diseases (CHD) should also be investigated for the involvement of hypertension with dyslipidemia [16]. In our study, blood laboratory analyses and anthropometric measurements were obtained from random participants of different ages and gender after their approval in the Specialized Polyclinic of Abha, Asir, South KSA. Blood serum samples were analyzed for the following measurements: (a) diabetic profiles, fasting blood glucose and Hb-A1C; (b) lipidemic parameters, total cholesterol (TC), triglycerides (TGC), HDLC, and LDLC; (c) Vitamin-D (Vit.D) and creatinine (Cr) for evaluating the hepatic and renal function; and (d) thyroid hormones; thyroid-stimulating hormone (TSH) and tetra-iodothyronine (F. T4) for evaluating the metabolic function. Anthropometric measurements included: (a) body mass index (BMI) for obesity and (b) blood pressure; systole, and diastole, for hypertension (HTN). Optimal cut-off values of the detected metabolic parameters were used as indicators of the cardiac risk factors: Castelli’s risk Factors; CRI-I and CRI-II, and atherogenic indices; AIP and AC, in addition to the triglyceride-glucose index (TyG) as an insulin-resistance marker. Different criteria were statistically investigated for studying the following issues: (a) metabolic profiles of the general population concerning gender, (b) MetS according to age and BMI in either sex in the general population, (c) cardiometabolic risk factors in each metabolic parameter according to cut-off values, (d) correlations and hierarchical clustering of the lipid profiles and cardiometabolic risk factors, and (e) prevalence of the cardiac risk and MetS in the mixed-hypercholesterolemic (HC) populations. 

The risky levels of metabolic profiles that trigger cardiovascular diseases, i.e., diabetes, dyslipidemia, and obesity, have to be clarified and studied at different ages of either sex for developing clinical guidelines of prevention and control of MetS. This study aimed to clarify the main component of MetS predisposing to CVD and to study the susceptibility of the MetS-adjusted CVD according to sex, age, and BMI in the studied populations. It also investigates the neighbor clustering of MetS components and clarifies interdigitate relations of metabolic profiles in the general, mixed-hypercholesterolemic, and diabetic populations.

## 2. Materials and Methods

### 2.1. Population and Studied Parameters

Parameters were studied for random participants in Specialized Polyclinic of Abha, Asir, Saudi Arabia, during the period from January 2020 to January 2021. The study was carried out on a total population of 648 participants, where 440 participants were recorded for the gender–180 males and 260 females–whereas 208 participants’ samples were referred to unrecorded gender. The different ages ranged from 15 to 98 years old (52.1 ± 1.1 Y.) (n = 242). All participants involved in the study excluded pregnant women, fractured, surgery-subjected, and cancer-diseased persons. Participants receiving treatment with drugs that could affect the pancreatic, liver, kidney, or thyroid function, i.e., lithium, amiodarone, methimazole, propylthiouracil, or thyroid therapy, were excluded. 

Serum samples of twelve-hour fasting participants were evaluated for the biochemical analysis, i.e., hemoglobin-A1C (HbA1C), fasting serum glucose (FSG), Vitamin-D (Vit.D), thyroid-stimulating hormone (TSH), free tetra-iodothyronine (F. T4), total cholesterol (TC), triglycerides (TGC), high-density lipoprotein cholesterol (HDLC), low-density lipoprotein-cholesterol (LDLC), hemoglobin (Hb), and creatinine (Cr). 

Anthropometric parameters were recorded, including body mass index (BMI) and blood pressure (Bp) parameters. The study was approved by the Ethics and Human Research Committee of King Khalid University (No. [ECM#2020-203]–[HAPO-06-B-001]). Informed consent was obtained from all participants for using their data. Laboratory measures were tested using Electrochemiluminescence assays (Siemens, Centaur XP).

### 2.2. Laboratory Assays, Data Curation, and Reference Ranges

All analyses were performed at the clinical pathology laboratory of the polyclinic. Blood markers were measured in serum samples of at least 12 h of a fasting period by using their specific kits. The oxidase method was used for assessment of glucose (Boehringer Mannheim, Mannheim, Germany) [18]. Hb-A1C was assayed using standardized reverse-phase chromatography by a fully automated Hb-A1C Menarini analyzer, based on reverse phase cation exchange–high-performance liquid chromatography (HPLC) [19]. The intraassay coefficient of variation was 0.65% at a mean of 4.89%, and the interassay coefficient of variation was 1.55% at a mean of 5.52%. TC and TGC were determined by enzymatic techniques according to commercial kits (Boehringer Mannheim, Germany) [20]. HDLC was directly measured by an enzymatic reaction using cholesterol oxidase according to the kits’ instructions (UniCel DxC 800; Beckman Coulter Inc., Pasadena, CA, USA) [20]. LDLC was estimated with the Friedewald formula when TGC was less than 400 mg/dL [20,21]. An immunodiagnostic assay was used for the determination of Vit. D concentrations depending on the assessment of 25(OH)-D regarding the commercial kits’ instructions (Immunodiagnostic-AG, Bensheim, Germany) [22]. Chemiluminescence immunoassay assay (CLIA) was used for measuring the serum concentrations of free thyroid hormones: T4 and TSH, using commercial kits (Architect^®^ CLIA, Abbott Diagnostic, Longford, Ireland) [23,24]. The enzymatic colorimetric method was used for the estimation of creatinine (Cr) by Siemens ADVIA Enzymatic reagent according to kits’ instructions (National Institute for Standards and Technology) [25]. 

Normal and risky reference ranges used for the tested physiological markers were as follows: blood sugar markers; normal A1C is below 5.7%, but 5.7–6.4% indicates prediabetes, and values ≥6.5% indicate diabetes [26]. FSG is normally between 70–100 mg/dL (3.9–5.6 mmol/L), and values between 100–125 mg/dL (5.6–6.9 mmol/L) indicate prediabetes, whereas those ≥126 mg/dL (7.0 mmol/L) refer to hyperglycemia [27]. Lipid profile markers: TC is normally <200 mg/dL, but 200–239 mg/dL is known as normally high (borderline), but risky values are those ≥240 mg/dL [28,29]. Normal ranges of TGC, HDLC, and LDLC were considered at: 150–200 mg/dL [29,30], 40–59 mg/dL [29], and 100–129 mg/dL [29,31], respectively. Vit. D is normally falling between 20–50 ng/mL [32]. Normal levels of thyroid function markers are: 0.35–4.5 uIU/mL and 12–20 pmol/L for TSH [33] and F. T4 [34,35], respectively. Cr was considered normal at 0.7–1.2 mg/dL in men and 0.5–1.0 mg/dL in women [36]. Hb is normally ≥13.5 g/dL in men and ≥12.0 g/dL in women [37]. 

### 2.3. Body Mass Index (BMI) and Blood Pressure (Bp: S/D)

The anthropometric parameters were measured, including body mass index (BMI) and blood pressure. The BMI was used for obesity determination based on the body weight and height of the participants, which were detected by using the approved formula of weight (kg)/[height (m)]^2^ [38]. Blood pressure (Bp) parameters, systole (S), and diastole (D) were recorded for hypertension (HTN) determination. BP was automatically measured by automatic cuff BP measurement devices based on oscillometry. Normal Bp is 120/80 based on S/D values. High blood pressure termed HTN is diagnosed when S/D is above 140/90 mmHg [39].

### 2.4. Statistical Analyses

Statistical analysis of data per metabolic parameters in the general population was performed to describe the percentages of normal vs. risky levels in male and female participants at different ages based on their normal concentration levels. All data were set as mean ± SEM and differences among groups were analyzed using Student’s *t*-test. Pearson correlations and the hierarchical dendrogram clustering of the parameters were performed using cross-linkages between the nearest neighbors’ groups. The area under the ROC–AUC curve with 95% CI was calculated to describe the sensitivity, specificity, and predictive cut-off values for the susceptibility of the different metabolic profiles to CVDs. One-way ANOVA was used to test the statistical differences among the different risk groups of each metabolic parameter; TC, TGC, HDLC, and LDLC. The Duncan-letter pattern was used by adding letters of significance on each bar. All statistics were performed using the statistical package for social sciences (SPSS) V. 20.0 (IBM Corp., Armonk, NY, USA) and Graph-Pad Prism Software V.3.0 (San Diego, CA, USA). The differences were considered significant at * *p* < 0.05.

## 3. Results

### 3.1. Metabolic Profiles of the General Population Concerning Gender

The general population was statistically describes as following: age (43.1 ± 0.63 Y.) (n = 242), BMI (32.8 ± 0.61 kg/m^2^) (n = 153) and gender (males (n = 180) and females (n = 260)). Metabolic profiles showed the following concentrations: A1C (6.83 ± 0.07%) (n = 480), FSG (137.2 ± 2.4 mg/dL) (n = 468), TC (215.8 ± 3.15 mg/dL) (n = 577), TGC (182.9 ± 3.5 mg/dL) (n = 557), HDLC (52.6 ± 1.6 mg/dL) (n = 475), LDLC (113.6 ± 1.8 mg/dL) (n = 430), Vit. D (31.94 ± 0.42 ng/mL) (n = 486), TSH (3.34 ± 0.13 uIU/mL) (n = 490), F. T4 (11.4 ± 0.31 pmol/L) (n = 392), Hb (12.79 ± 0.11 g/dL) (n = 648), and systolic vs. diastolic blood pressure (131.8 ± 1.14 vs. 71.9 ± 0.64 mm Hg) (n = 216), and Cr (0.93 ± 0.52 mg/dL) (n = 473). According to metabolic profiles and BMI, MetS in the general population showed higher risk levels for diabetes (T2DM), hypothyroidism (HT), and obesity—69.4, 85.5, and 92.2%, respectively. Other risky profiles above 50% were ordered as: 56.2 and 53.3% for hypovitaminosis-D and low LDLC, respectively (Table 1).

**Figure 1:**
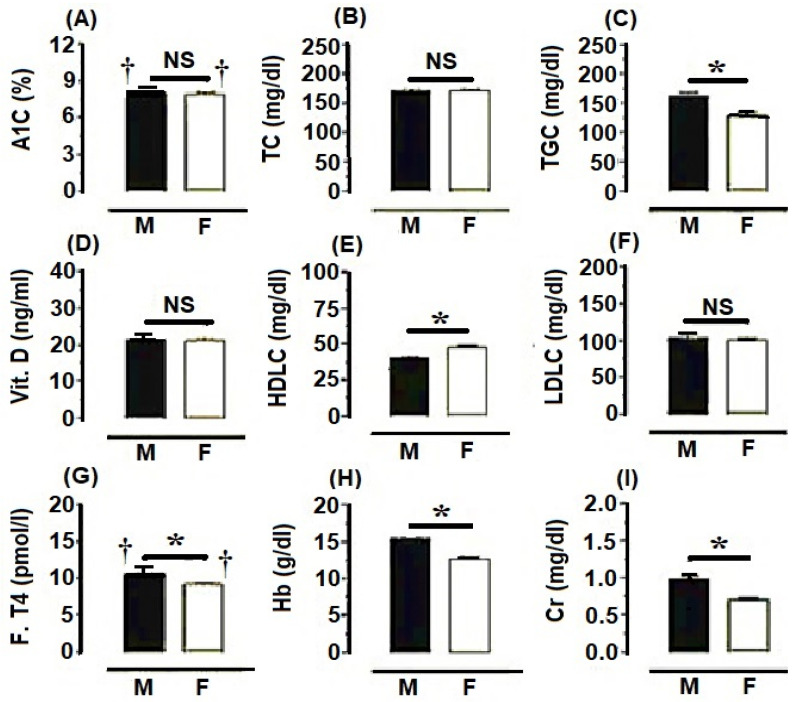
Characteristics of metabolic profiles in both male (M) and female (F) participants of the general population in Asir, South KSA. All data were expressed as mean ± SEM. Asterisk (*) denotes significant difference (*p* < 0.05) between males and females. The sign (†) denotes risky concentration level of the parameter. NS denotes a nonsignificant difference. Other explanations were given in Table 1.

Metabolic profiles of men and women participants in the general population are shown in Figure 1. Metabolic profiles showed variations in men and women. Women showed a higher incidence of hypothyroidism and diabetes compared to men. However, the incidence of hypovitaminosis-D and risky low LDLC were higher in men rather than women. General profiles of several metabolic parameters in men were significantly higher compared to each respective profile in women (TGC: 161.1 ± 6.8 vs. 128.6 ± 4.85 mg/dL, F. T4: 10.5 ± 0.46 vs. 9.2 ± 0.15 pmol/L, Hb: 15.4 ± 0.12 vs. 12.7 ± 0.10 g/dL, and Cr: 0.99 ± 0.03 vs. 0.70 ± 0.02 mg/dL, respectively) (*p* < 0.05). On the other hand, the general profile of HDLC was significantly higher in women rather than men (47.9 ± 1.04 vs. 40.2 ± 0.92 mg/dL, respectively) (*p* < 0.05) (Figure 1).

### 3.2. Metabolic Syndrome (MetS) According to Age with Respect to Gender in the General Population

Prevalence of risky MetS in both sexes according to age: less and more than 40 Y. old are shown in Table 2. Males <40 Y. were the most participants suffering from hypothyroidism (F. T4: 9.96 ± 0.52 pmol/L), but those elders of ≥40 Y. were mostly suffering from T2DM (A1C: 8.6 ± 1.20%) and dyslipidemia, including low HDLC (37.4 ± 2.7 mg/dL) and LDLC (99.8 ± 18.6 mg/dL). Females <40 Y. were deficient in Vit. D (16.13 ± 1.7 ng/mL) and Hb (11.34 ± 0.4 g/dL). However, females ≥40 Y. showed a risk of T2DM (A1C: 6.97 ± 0.85%). Hypothyroidism was affecting both young and old females: F. T4; 8.45 ± 0.25 vs. 9.75 ± 0.70 pmol/L, respectively. 

### 3.3. MetS According to BMI in the General Population

Characteristics of the general population according to BMI: overweight and obese are shown in Table 3. The ages recorded for obesity were significantly higher than those of overweight (60.0 ± 1.6 vs. 54.0 ± 2.7 kg/m^2^, respectively) (*p* < 0.05). The mean BMI recorded for overweight participants was significantly different than that recorded in obese ones (27.0 ± 0.2 vs. 37.0 ± 0.7 kg/m^2^) (*p* < 0.05). MetS showed paralleled prevalence of risky profiles in both overweight and obese participants. Obese participants showed a higher incidence of metabolic risk compared to those of overweight, including diabetes (A1C: 97.8 vs. 93.3%), hypertension (HTN: 41.1 vs. 15.6%), anemia (low Hb: 23.0 vs. 14.0 g/dL), low LDLC (60.0 vs. 55.0%), hypothyroidism (F. T4: 83.1 vs. 82.8%), hypovitaminosis-D (Vit.D: 65.6 vs. 64.3%) and hypocreatinemia (low Cr: 42.7 vs. 24.5%) per each respective population. Incidence of dyslipidemia in the overweight participants was riskier than in the obese: hypercholesterolemia (TC: 8.5 vs. 6.8%), higher TGC (27.3 vs. 16.9%), higher LDLC (25.0 vs. 7.5%) and low HDLC (51.0 vs. 40.2%) per each respective population. Finally, the incidence of hypercreatinemia was significantly higher in the overweight population rather that of obesity (24.5 vs. 18.4%, respectively).

### 3.4. Cardiometabolic Risk Factors and Insulin-Resistance Marker

ROC–AUC calculated for cut-off values of cardiometabolic risk factors in MetS relevant parameters are shown in Table 4. Dyslipidemic profiles: TC, TGC, HDLC, and LDLC showed the highest area under the curve (AUC), cut-off values, and sensitivity (SEN) of Castelli’s risk factors: RI-I/CRI-II and atherogenic indices: AIP/AC. Participants with hypercholesterolemia (HC) and lower HDLC showed the most sensitive profiles in ROC–AUC for the cardiac risk factors: CRI-I. However, TyG showed the highest significant AUC, cut-off, and SEN to TGC (0.908, 9.36 and 0.90, respectively), FSG (0.779, 0.920 and 0.66, respectively) and A1C (0.684, 0.923 and 0.53, respectively) (*p* < 0.05). 

Participants affected with HTN showed significant cut-off values of CVDs as: 3.71, 0.57, and 2.71 for CRI-I, AIP, and AC, respectively (*p* < 0.05), recording high sensitivity above 0.69 (*p* < 0.05). All the cardiometabolic risk factors significantly correlated with age in a positive pattern showing the highest correlation with TyG; 0.347. However, hypovitaminosis-D showed a nonsignificant correlation with those risk factors.

**Figure 2:**
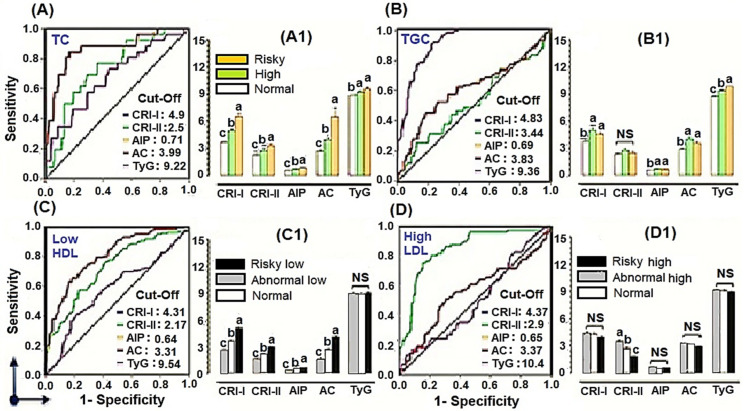
AUC–ROC curves of positive vs. negative incidence of CVDs in the dyslipidemic profiles via their sensitivities to the cardiometabolic risk factors and cut-off values (**A**–**D**). AUC–ROC above 0.6, 0.7, and 0.8 but not those less than 0.6 were considered for their cut-off values. Risk indices in normal vs. abnormal levels of the lipidemic profiles are shown in (**A1**,**B1**,**C1**,**D1**). Differences between groups were considered significant at *p* < 0.05. NS: non-significant. Letters on bars; a, b, c, and d denote significant difference among groups.

AUC–ROC curves for susceptibility of the dyslipidemic population to CVDs are shown in Figure 2. Cut-off values of the cardiac risk factors were calculated depending on AUCs and sensitivities of the lipid parameters. i.e., cut-off value and AUC of CRI-I for TC were 4.99 and 0.864, respectively, which means that participants with CRI-I ≥ 4.99 were susceptible to CVDs, but those lower <4.99, were not susceptible. Consequently, Figure 2 (A1,B1,C1,D1) compared the mean risk factors of CRI-I, CRI-II, AIP, AC, and TyG in the different levels of each metabolic parameter and confirmed that the abnormal risk levels were directly proportional to the participant susceptibility to CVDs. Moreover, according to the TyG marker, Figure 2 showed that insulin resistance could be associated with the risky TC and TGC but not associated with HDLC and LDLC. As shown in Figure 2, the risk factors: CRI-I, CRI-II, AC, and AIP, confirmed the incidence of CVDs, especially in those with risky profiles; for example, TC; ≥240 mg/dL (Figure 2A1), TGC; ≥ 200 mg/dL (Figure 2B1), low HDLC; <40 mg/mL (Figure 2C1), and high LDLC; ≥130 mg/dL (Figure 2D1) were more susceptible to the cardiovascular diseases.

### 3.5. Correlations and Hierarchical Clustering of the Lipid Profiles and Cardiometabolic Risk Factors

**Figure 3:**
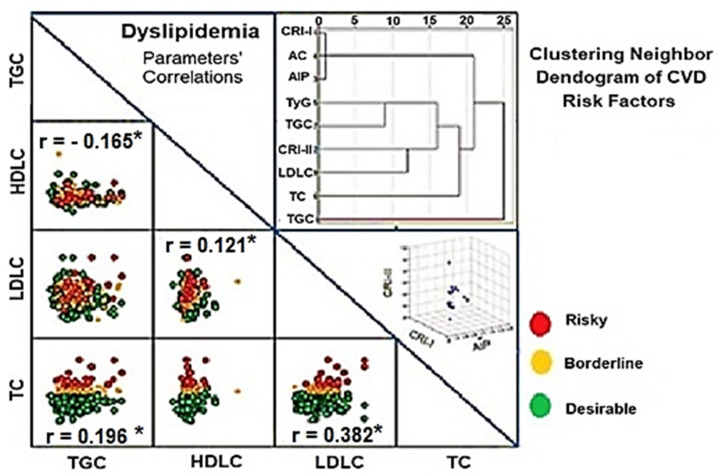
Pearson correlations (r) of lipid profiles: TC, TGC, HDLC, and LDLC as the dyslipidemic parameters, the main predisposing factor of CVD. The hierarchical dendrogram showed a clustering analysis of the cross-linkages between the nearest neighbor cardiometabolic risk factors and lipid profiles. Risky, borderline, and desirable levels of hypercholesterolemia, hypertriglyceridemia, HDLC, and LDLC, are shown in Table 1. Asterisk (*) denotes a significant difference at *p* < 0.05.

Pearson correlation matrix of the lipid profiles: TC, TGC, HDLC, and LDLC, and a dendrogram of the hierarchical cluster analysis for the cross-linkages of cardiometabolic risk factors and those lipid profiles are shown in Figure 3. TC significantly correlated with both TGC (R = 0.196) and LDLC (R = 0.382), but not HDLC. However, TGC showed a significant inverse proportion to HDLC (R = −0.165) and LDLC significantly correlated with HDLC in a positive pattern (R = 0.121) (*p* < 0.05). The dendrogram showed three main clusters: the first neighbor’s cluster included CRI-I, AC, and AIP, the second cluster showed the presence of linkage between the TGC and TyG, and finally, the third cluster showed the nearest linkage between CRI-II and LDLC. 

### 3.6. Prevalence of Cardiac Risk and MetS in the Mixed-Hypercholesterolemic (HC) Populations

**Figure 4:**
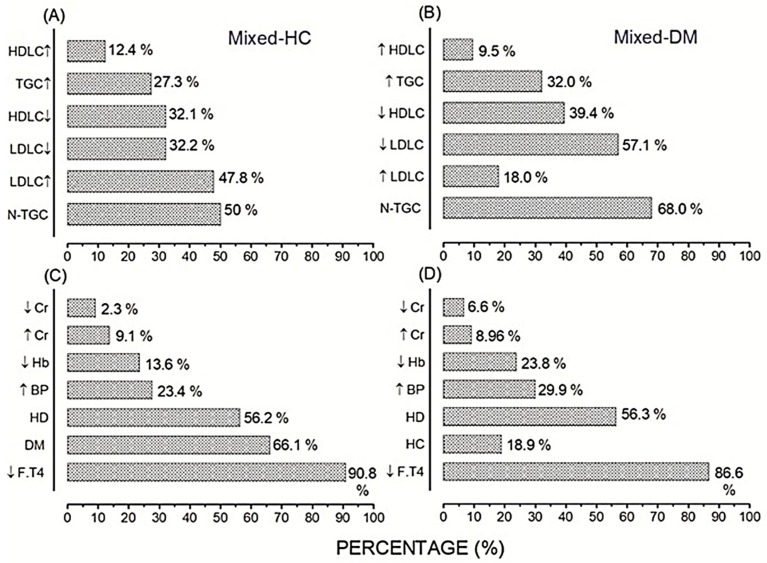
Prevalence of MetS in the mixed–HC (**A**,**C**) compared to those in the mixed-diabetic (**B**,**D**) populations. N-TGC denotes the normal triglycerides. Arrows indicated the higher and lower levels of TGC, HDLC, LDLC (**A**,**B**), F. T4, BP, Hb, and Cr (**C**,**D**).

Mixed-hypercholesterolemia referred to dyslipidemic participants mainly affected with hypercholesterolemia and abnormal profiles of lipidemic constituents: TGC, LDLC, HDLC (Figure 4A), in addition to the other MetS components in different percentages, i.e., hypothyroidism (HC–HT) (90.8%), diabetes (HC–DM) (66.1%), hypovitaminosis-D (HC–HD) (56.2%), hypertension (HC–HTN) (23.4%), anemia (HC–anemic Hb) (13.6%), hypercreatinemia (9.1%), and hypocreatinemia (2.3%) (Figure 4C). However, 10.7 vs. 89.3% of the total lipidemic population (n = 112) were participants suffering from hypercholesterolemia (only) versus those aggravated with the other MetS components (mixed–HC). Participants with DM showed abnormal lipidemic profiles with higher levels of TGC (Figure 4B). Hypothyroidism, hypovitaminosis-D, hypertension, anemia, and creatinemia showed respective high incidences in the mixed DM participants (Figure 4D). 

The prevalence of CVDs in the mixed–HC population was calculated according to the mean incidence of the risky cardiometabolic factors, exceeding their cut-off, as follows: CRI-I plus CRI-II, AC plus AIP, in addition to TyG (Table 5). Thus, the HC–HT population showed a mean risk incidence of CVDs as: 54.1, 50.4, and 54.0%, respectively. Susceptibility to CVDs in HC–MetS components was variable. HC mixed with hypertension (HC–HTN) was the most susceptible to CVDs followed, in order, by diabetes (HC–DM), hypothyroidism (HC–HT), and hypovitaminosis-D (HC–HD) (Table 5). They showed mean incidences as: 54.4, 51.7, and 52.0% for HC–DM; 49.0, 46.5 and 42.6% for HC–HD; 64.9, 58.2 and 69.6% for HC–HTN; and 49.6, 37.6 and 39.1% for HC–anemic Hb.

Moreover, HC–males showed a higher incidence of Castelli’s risk factors, atherogenic indices, and TyG, rather HC–females: 56.0, 64.7, 56.3 vs. 44.1, 32.4, and 39.0%, respectively. Further, HC participants aged ≥40 Y. showed a higher incidence of the risk factors compared to those < 40 Y.: 64.0, 64.0, 70.0 vs. 58.3, 33.3, and 60.0%, respectively. Those cardiac risk factors: CRI-I, AC, and AIP showed a higher incidence of CVD risk in HC–overweight rather than HC–obese participants; 78.6 vs. 66.7%, respectively. However, CRI-II and TyG showed higher incidence of CVD risk in HC–obese participants rather than HC–overweight: 66.7 vs. 64.3% and 100.0 vs. 71.4%, respectively (Table 5). 

## 4. Discussion

The steady socioeconomic changes in Saudi Arabia show variations in the diet with a marked shift to a sedentary urban lifestyle. It was linked and paralleled to an elevation in metabolic abnormalities worldwide. Our study revealed various metabolic changes between both sexes before and after the age of 40. MetS is a research point of interest for several years as it affects more than 25% of the total adult population in the world due to its direct relation to CVDs [10]. This study was the first in Saudi Arabia to investigate the susceptibility of CVDs in different populations, depending on the cardiometabolic risk factors in the MetS’-related criteria: dyslipidemia, diabetes/insulin resistance, hypertension, anemia, hypothyroidism, hypovitaminosis-D, and creatinemia, and clarifies the prevalence of MetS/CVDs in either sex before and after the age of 40 [40]. 

It was an endeavor to elucidate the prevalence of risk levels of MetS/CVDs in the general population in Asir, Saudi Arabia at different ages of either sex, with a special focus on the mixed–HC population. Dyslipidemia and diabetes were the most components of MetS detected in the studied general population [41]. Consistent with a previous study [42], participants with HC and T2DM were the most susceptible patients to CVDs. Our findings show that 66.1% of the mixed-HC participants were diabetic, but only 18.9% of mixed-diabetics were HC, which is in agreement with a recent study reporting that dyslipidemia is highly prevalent among diabetic patients [43]. Although both sexes showed a risk of dyslipidemia and diabetes, variable risk levels were also detected for other metabolic criteria, i.e., hypothyroidism, hypovitaminosis-D, and anemia, which were higher in women rather than males. This finding was consistent with a previous study proving the sex specificity of MetS to be higher in women rather than men with a prevalence of 29 vs. 23%, respectively [44,45]. 

Although women showed a higher prevalence of MetS and dyslipidemic obesity than men, the mean Castelli’s (CRI-I/CRI-II) and atherogenic (AC/AIP) risk factors with a further insulin-resistance marker (TyG) were higher in the mixed–HC men rather than women, and further, the lipidemic profiles showed higher TGC, but low HDLC, in elder males (≥40 Y.) than females, which supported the previous report in our city [46]. Those findings support that men are more susceptible candidates for CVDs rather than women. Several studies proved a significant association between hypertriglyceridemia and the risk of CVDs [47].

Furthermore, CVD is associated with lipid accumulation in the human body that is varied between both sexes and their physiological condition, i.e., premenopausal women are more susceptible to peripheral obesity with subcutaneous fat deposition, but men and postmenopausal women are more prone to central or android obesity [48]. Particularly, CVD was found to associate with the visceral and peripheral adipocytes which are different in their lipolytic response to insulin, adrenergic/angiotensin stimulation, and sex hormones. Visceral adipocytes are the origin of free fatty acids infiltrated with adipokines [49], which are markedly elevated in obesity and diabetes [49]. Those cytokines stimulate insulin resistance, atherogenic changes, dyslipidemia, high blood pressure and so susceptibility to CVD, especially in women [50]. Visceral adiposity lacks adiponectin, a tissue-specific hormone that stimulates glucose use and fatty acid oxidation in muscles, promoting insulin sensitivity in the liver and reducing hepatic glucose output [51,52]. 

According to NCEP–ATP III and IDF, the prevalence of MetS was 83% for men and 86% for women and increased with age in both sexes [53], which confirmed our findings that women showed higher susceptibility to MetS than men. Further, our study stated that MetS increased with ages ≥40 Y. in both sexes. It also presented a high prevalence of T2DM and hypothyroidism in men <40 Y. compared to women of the same ages. On the other hand, incidences of hypovitaminosis-D and anemia were more prevalent in women <40 Y. than in men. CVDs mortality and stroke were independent with age in men, but in women, stroke was found to increase with age [54]. It could be attributed to increased BP occurring in women after menopause which causes the sudden decline of the endothelial function in CVD [55,56]. However, elder men showed less elevation of BP and were also associated with less prevalence of MetS at old ages [56]. 

Recently, several reports studied MetS prevalence for country variation, including Germany [55], Norway [57], and Greece [58], which revealed MetS prevalence as 9–16% in males <40 Y. and 34–45% in males ≥40 Y., whereas in women <40 Y. was 5–8%, and women ≥40 Y. was 35–46%, confirming the susceptibility of MetS to age in either sex. It coincided with our findings in Saudi Arabia, as MetS was more susceptible not only to age, where it was higher in elders rather youngers, but also to sex, being higher in women rather than men. Genetics, lifestyle, and environmental habits are important factors affecting MetS as well [59]. Accordingly, the higher incidence of MetS recorded in the general population of Saudi Arabia could be attributed to developing habits of increased consumption of unhealthy junk food, high calories of sugar, and fatty foods, and mainly in adult women rather than men as previously studied in Jeddah city, KSA [60]. Further, the alarming increment of tobacco smoking and lack of exercise among adult Saudi people [61] should not be neglected as an important candidate factor for increasing the incidence of MetS.

MetS develops several metabolic hazards aggravating serious forms of CVD, such as atherosclerosis, CHD, and stroke. A previous genetical study confirmed an association of dyslipidemia with apolipoprotein A5 gene-1131T/C polymorphism as a powerful promotor of CHD [62]. This gene was detected in the characteristic forms of dyslipidemia: high levels of TGC and decreased levels of HDL-C [63]. Moreover, the high prevalence of MetS in women was attributed to abdominal obesity and insulin resistance in association with reduced physical activity and/or polycystic ovarian syndrome [64]. Additionally, high systolic blood pressure (SBP) was found to correlate with hyperinsulinemia in T2DM and other bad habits such as smoking and alcoholism [65].

Abnormal fat distribution is an important predisposing factor of MetS in either sex [66]. The worldwide prevalence of obesity doubled during the period from 1980 to 2014. In 2014, the WHO recorded that 38% of men vs. 40% of women were overweight and 11% of men vs. 15% of women were obese [67,68]. Those reports coincided with ours because three of each five men were overweight, but three of each four women were obese. Body mass index (BMI) has been used for indirect evaluation of MetS’ risk, according to which, the population was classified into normal weight (BMI: <25 kg/m^2^), overweight (BMI: 25–30 kg/m^2^), and obese (BMI: >30 kg/m^2^) [69]. In our findings, MetS was prevalent in the obese population with BMI; 37.0 ± 0.7 kg/m^2^, rather those overweight with BMI: 27.0 ± 0.2 kg/m^2^. Obesity was more prominent in several MetS categories, like hypothyroidism, diabetes, hypovitaminosis-D, hypertension, anemia, low LDLC, and hypercreatinemia. Previous studies showed that overweightness and obesity directly contribute to CVDs [70,71], but others reported that MetS and CVD were independent of high BMI in aged men [69].

Not only has dyslipidemia been considered the most linked factor with MetS and CVD [72], but also hypovitaminosis-D [73]. It is worth mentioning that dyslipidemia has been monitored by low HDLC, high TGC, and high LDLC, which were considered the main risk indicator for CVD [74]. Although prospective studies indicated an enhanced risk of CVD when the circulating 25-hydroxyvitamin-D was below 25 nmol/l, regarding the triglyceride-lowering effect of Vit. D [73]. Young Saudi women less than 40 Y. old that showed a deficiency of Vit. D is in agreement with several previous reports which attributed this deficiency to the lack of exposure to sunlight, staying indoors, and veiling [75,76]. A large portion of vitamin D3 is converted into the active form from exposure to sunlight which is typically prevented by the traditional clothing worn by Saudi women [77]. Although the present findings showed nonsignificant cardiometabolic risk among women suffering from Vit. D deficiency, we strongly agree with a previous call for a national strategy to control the hypovitaminosis-D crisis in KSA [77]. 

Therefore, the atherogenic index of plasma (AIP) and Castelli’s indices-I and II are biomarkers for lipid atherogenic risk and assessment of CVD risk depending on the lipid profiles. Consequently, CRI-I and II were elevated in MetS combined with dyslipidemia [78]. AIP is an atherogenic marker for the relevance of protective HDLC and atherogenic TGC lipoprotein and was considered a powerful predictor of atherosclerosis and CHD [79]. The present study agreed with the previous reports focused on the elevation of AIP with MetS [80]. However, other studies stated a sex variation in AIP showed elevation in females more than males [81]. From a physiopathological view, AIP elevation indicated higher TGC and lower HDLC, which in turn predispose them to the development of atheromatous plaque [82,83] and are considered characteristic factors of diabetic dyslipidemia [84]. Both biomarkers’ disturbances result in competition for glucose transport through the cell membrane, glucose oxidation, and glucose transporters ending with insulin resistance and downregulation of insulin receptors on adipocytes [85]. According to our findings, the increased MetS in the general population of Saudi Arabia could be attributed to the changed environmental habits mainly the increased consumption of junk food and tobacco smoking as stated by previous social health reports. 

## 5. Conclusions

MetS affects women more than men, but the possibility of cardiac risk was higher in mixed-hypercholesterolemic males rather than females, with an increased incidence in elders rather than youngers, and in overweight rather obese. The study also showed clear susceptibility of females to anemia, diabetes, and hypovitaminosis-D rather than males. Moreover, 66.1% of the mixed-HC population were diabetic participants, but 18.9% of the mixed-DM population were hypercholesterolemic. A complementary study is required in future to investigate the correlation between the increased prevalence of MetS and CVD and the environmental habits in Saudi Arabia among the population.

## Figures and Tables

**Table 1:** Characteristics of the risky MetS according to gender in the general population. Metabolic profiles include: A1C and FSG for T2DM, TC, TGC, HDLC, and LDLC for dyslipidemia, Vit. D for hypovitaminosis-D, TSH and F. T4 for hypothyroidism, Hb diagnosed anemia and Cr for renal function, in addition to the blood pressure parameters of systole and diastole. BMI was also detected. † denotes risky levels: L; low, H; high. M; male, and F; female. All data were presented as mean ± SEM. Differences between overweight and obese groups were considered significant at * *p* < 0.05–NS denotes a nonsignificant difference.

MetS-Related Criteria	† Parameters		Characteristics of the General Population According to Gender								*p* Value
			Risk Profile	%	† Males	N	%	† Females	N	%	
Body Mass Index–BMI (kg/m^2^)			33.7 ± 0.61	92.2	27.73 ± 0.27	3/5	60.0	33.27 ± 4.26	3/4	75.0	0.26
Diabetes T2DM	† A1c ≥ 6.4 (%)		9.2 ± 0.11	69.4	9.51 ± 0.22	86/144	59.7	9.13 ± 0.21	127/196	64.8	0.11
	† FSG ≥ 125 (mg/dL)		212 ± 5.1	53.2	229.1 ± 12.3	64/133	48.1	210.9 ± 8.40	85/190	44.7	0.10
Dyslipidemia	† TC: ≥ 240 (mg/dL)		280.0 ± 7.1	5.20	293.8 ± 16.8	10/179	5.60	279.0 ± 9.36	10/261	3.83	0.23
	† TGC: ≥ 200 (mg/dL)		274.5 ± 6.3	21.2	280.7 ± 9.92	50/177	28.3	256.2 ± 10.7	42/247	17.0	0.08
	HDLC mg/dL	L.: <40	32.6 ± 0.4	37.7	32.2 ± 0.81	66/135	48.9	33.8 ± 0.91	55/202	27.2	0.19
		H.: >59	77.2 ± 3.9	9.10	68.0 ± 3.34	6/135	4.40	69.8 ± 2.53	22/202	10.9	0.72
	LDLC mg/dL	L.: <100	73.4 ± 1.3	53.3	68.6 ± 2.65	66/126	52.4	72.0 ± 2.20	89/179	49.7	0.32
		H.: >129	156.1 ± 3.1	20.9	157.4 ± 5.79	37/126	29.4	151.0 ± 3.10	37/179	20.7	0.32
Hypovitaminosis-D (Vit. D: ng/mL)		L.: <20	13.49 ± 0.3	56.2	13.9 ± 0.74	77/144	53.5	13.16 ± 0.36	138/250	55.1	0.16
		H.: >50	53.5 ± 0.47	2.26	53.4 ± 0.47	7/144	4.86	53.70 ± 1.11	4/250	1.60	0.39
Hypothyroidism (HT)	TSH uIU/mL	L.: <0.4	0.24 ± 0.11	3.30	0.11 ± 0.04	8/174	4.60	0.15 ± 0.03	10/321	3.12	0.21
		H.: >5.0	7.48 ± 0.23	19.9	7.50 ± 0.54	20/174	11.50	7.75 ± 0.31	77/321	23.9	0.36
	F.T4 pmol/L	L.: <12.0	8.79 ± 0.09	85.5	8.81 ± 0.25	71/91	78.0	8.71 ± 0.12	186/212	87.7	0.34
		H.: ≥20.0	22.2 ± 0.00	0.26	22.2 ± 0.00	1/91	1.10	----	----	----	----
Anemia	† Hb: <13.5 M-12.0 F. (g/dL)		10.9 ± 0.12	24.9	11.9 ± 0.37	17/190	8.95	10.5 ± 0.12 *	101/280	36.1	**<0.0001**
Creatinemia	Cr mg/dL	L. <0.7 M.–0.5 F.	0.57 ± 0.11	10.2	0.58 ± 0.02	18/183	9.80	0.41 ± 0.01 *	30/290	10.4	
		H. >1.2 M.–1.0 F.	1.44 ± 0.05	10.4	1.70 ± 0.17	20/183	10.9	1.30 ± 0.07 *	29/290	10.0	**0.0185**
Hypertension (HTN)	† Systole: ≥ 140 mm Hg		152.9 ± 1.6	25.5	148.0 ± 3.52	9/27	33.3	143.6 ± 1.1	13/57	22.8	0.09
	† Diastole: ≥ 90 mm Hg		75.0 ± 1.39	25.5	80.1 ± 3.26	9/27	33.3	76.2 ± 1.9	13/57	22.8	0.14
	Blood Pressure		153/75	26.0	148/80	9/27	33.3	144/76	13/57	22.8	--

Bold *p* values denote significance.

**Table 2:** Prevalence of the metabolic syndrome profiles in males and females of the general population with respect to age: below 40 years old (>40 Y.) or equal/after that age (≥40 Y.). † denotes risky levels. All data were presented as mean ± SEM. Mean differences of each parameter between both ages were analyzed by Students’ *t*-test and considered significant at * *p* < 0.05–NS, denoting a non-significant difference. Elders (≥40 Y.) were susceptible to diabetes in both men and women. Ages <40 Y. were susceptible to hypothyroidism in males, but susceptible to hypovitaminosis-D, hypothyroidism, and anemia in young females.

Gender	Males					Females				
Age	<40 Y.	N	≥40 Y.	N.	*p* Value	<40 Y.	N	≥40 Y.	N.	*p* Value
	31.9 ± 1.5	14	61.8 ± 2.30 *	20	**<0.0001**	31.9 ± 0.83	36	59.6 ± 2.70 *	33	**<0.0001**
A1C (%)	5.5 ± 0.09	9	† 8.6 ± 1.20 *	8	**0.015**	6.00 ± 0.00	2	† 6.97 ± 0.85	15	---
FSG (mg/dL)	102.7 ± 1.1	5	† 145.3 ± 29.4	6	0.223	87.4 ± 3.56	10	† 139.1 ± 44.1	6	0.147
TC (mg/dL)	183.2 ± 10.8	9	167.2 ± 16.10	19	0.523	175.8 ± 9.6	11	179.7 ± 40.5	13	0.932
TGC (mg/dL)	113.1 ± 12.8	9	132.7 ± 22.40	19	0.569	95.3 ± 21.3	9	129.5 ± 43.9	11	0.522
HDLC (mg/dL)	41.6 ± 2.47	9	† 37.4 ± 2.70	19	0.337	50.2 ± 4.03	11	47.3 ± 6.9	13	0.733
LDLC (mg/dL)	141.7 ± 4.6	7	† 99.8 ± 18.60	16	0.158	104.2 ± 9.7	12	109.4 ± 28.9	16	0.882
Vit.D (ng/mL)	32.57 ± 7.1	6	25.0 ± 4.40	17	0.385	† 16.13 ± 1.7	15	21.65 ± 2.6	27	0.146
F. T4 (pmol/L)	† 9.96 ± 0.52	14	12.4 ± 1.65	20	0.238	† 8.45 ± 0.25	35	† 9.75 ± 0.70	33	0.078
Hb (g/dL)	14.89 ± 0.7	10	15.4 ± 0.60	16	0.592	† 11.34 ± 0.4	28	12.75 ± 0.50 *	29	**0.033**
Cr (mg/dL)	0.95 ± 0.05	12	1.04 ± 0.02	13	0.099	0.64 ± 0.03	19	0.83 ± 0.10	20	0.083
Systole (mmHg)	133.7 ± 3.1	12	134.9 ± 5.50	18	0.869	120.5 ± 2.5	29	129.3 ± 4.35	28	0.082

Bold *p* values denote significance.

**Table 3:** Characteristics of the general population according to body mass index (BMI): overweight (n = 57) and obese (n = 90) showing the incidence of risky parameters in each category. Asterisk (*) denotes significant difference (*p* < 0.05) between overweight and obese groups. NS means nonsignificant difference between both groups. † Risky Levels. O.W: Overweight.

† Parameters and MetS		Characteristics of Population According to BMI (kg/m^2^)							*p* Value
		Reference Risk	O.W: 25–29.9	N	%	Obese: ≥30	N	%	
Abnormal BMI (kg/m^2^)		>25	27.0 ± 0.2	57/153	37.3	37.0 ± 0.7 *	90/153	58.8	**0.0001**
† High Systolic BP (mm Hg)		≥140	157.1 ± 5.7	8/45	17.8	153.1 ± 1.9	35/83	42.2	0.204 ^NS^
† High Diastolic BP (mm Hg)		≥90	90.0 ± 0.0	3/45	6.7	NA	--	--	----
Hypertension (S/D)		≥140/90	164/86	7/45	15.6	154/72	34/83	41.1	----
Anemia–† Low Hb (g/dL)		<13.5 M–<12.0 F	10.5 ± 0.50	7/50	14.0	11.1 ± 0.17	21/91	23.0	0.077 ^NS^
DM–† A1c (%)		>5.7	8.26 ± 0.24	42/45	93.3	9.01 ± 0.22 *	88/90	97.8	**0.019**
DM–† FSG (mg/dL)		>125	180.8 ± 10.8	29/48	60.4	206.0 ± 9.1	62/88	70.5	0.051 ^NS^
HC–† High T.C (mg/dL)		≥240	289.6 ± 26.8	4/47	8.50	270.2 ± 11.6	6/88	6.80	0.236 ^NS^
HC–Borderline (mg/dL)		200–239	211.0 ± 4.8	8/9	88.9	NA	0	0.0	----
† High T.G.C (mg/dL)		>200	279.5 ± 14.1	12/44	27.3	293.3 ± 16.4	14/83	16.9	0.268 ^NS^
HDLC (mg/dL)	† Low	<40	41.0 ± 1.6	23/45	51.0	47.0 ± 2.4 *	35/87	40.2	**0.035**
	† High	>59	64.4 ± 4.7	3/45	6.70	87.5 ± 10.6	12/87	13.8	0.155 ^NS^
LDLC (mg/dL)	† Low	<100	70.2 ± 3.1	22/40	55.0	80.8 ± 1.3 *	48/80	60.0	**<0.0001**
	† High	>129	165.2 ± 15.1	10/40	25.0	168.6 ± 4.1	6/80	7.50	0.434
Vit.D (ng/mL)	† Low	<20	13.5 ± 1.3	18/28	64.3	14.5 ± 0.7	40/61	65.6	0.232
	† High	>50	52.4	1/28	3.60	52.4	1/63	1.60	----
TSH (uIU/mL)	† Low	<0.3	0.20 ± 0.00	2/34	5.9	NA	--	--	----
	† High	>5.0	7.63 ± 1.18	7/34	20.6	6.49 ± 0.34	13/63	20.6	0.122
F.T4 (pmol/L)	† Low	<12	8.7 ± 0.3	24/29	82.8	9.2 ± 0.2	49/59	83.1	0.081
	† High	≥20	NA	--	--	NA	--	--	----
Cr (mg/dL)	† Low	<0.7 M–<0.5 F	0.57 ± 0.03	12/49	24.5	0.56 ± 0.01	38/89	42.7	0.342
	† High	>1.2 M–>1 F	1.36 ± 0.06	12/49	24.5	1.37 ± 0.13	16/87	18.4	0.475

Bold *p* values denote significance.

**Table 4:** AUC–ROC above 0.6, 0.7, and 0.8 were used for cut-off values of CVDs’ susceptibility and sensitivity (SEN) in MetS. Castelli’s risk index: CRI-I and CRI-II, atherogenic index in plasma (AIP), atherogenic coefficient (AC), and triglyceride-glucose index (TyG). MetS parameters include TC, TGC, HDLC, A1C, FSG, F. T4, Vit.D, Hb, Cr, and BP, in addition to age >60 Y. Pearson correlations (R) were calculated for metabolic syndrome and risk factors. Asterisk (*) for AUC means acceptable values of discrimination between the positive and negative affections; 0.5 = no discrimination, 0.6–0.7 = poor, 0.7–0.8 = good, 0.9–1.0 = excellent. The asterisk of Pearson correlations denotes significant difference at *p* < 0.05.

Metabolic Predictors		Serum Biomarkers in Different MetS’ Populations												Age
		Dyslipidemia				DM		HT	HD	Anemia	High BP (S/D)	Creatinemia		
		TC	TGC	Low HDLC	High LDLC	A1C	FSG	F.T4	Vit. D	Low Hb		High Cr	Low Cr	
CRI-I	AUC	0.864 *	0.608 *	0.805 *	0.560	0.494	0.525	0.498	0.485	0.416	0.627 *	0.526	0.583	0.604
	Cut-off	4.99	4.83	4.31	4.37	---	3.87	---	---	---	3.71	3.67	3.18	2.59
	SEN	0.85	0.45	0.65	0.51	---	0.52	---	---	---	0.69	0.63	0.93	0.90
	r	0.569 *	0.292 *	−0.592 *	0.143 *	−0.035	0.078	0.064	−0.005	0.125 *	−0.075	0.049		0.282 *
CRI-II	AUC	0.735 *	0.522 *	0.725 *	0.848 *	0.408	0.470	0.486	0.454	0.478	0.609 *	0.536	0.462	0.580
	Cut-off	2.47	3.44	2.17	2.92	---	---	---	---	---	2.37	4.19	---	1.33
	SEN	0.77	0.26	0.76	0.76	---	---	---	---	---	0.55	0.20	---	0.82
	r	0.198 *	0.115 *	−0.366 *	0.754 *	−0.106 *	−0.009	0.087	.024	0.085	−0.056	0.132 *		0.230 *
AIP	AUC	0.865 *	0.608 *	0.807 *	0.559	0.495	0.527	0.499	0.485	0.415	0.631 *	0.524	0.583	0.605
	Cut-off	0.71	0.69	0.64	0.65	---	0.86	---	---	---	0.57	0.61	0.51	0.42
	SEN	0.85	0.44	0.65	0.49	---	0.53	---	---	---	0.69	0.53	0.93	0.90
	r	0.563 *	0.258 *	−0.729 *	0.131 *	−0.022	0.079	0.064	0.040	0.053	−0.074	0.052		0.280 *
AC	AUC	0.864 *	0.608 *	0.805 *	0.560	0.494	0.525	0.498	0.485	0.416	0.627 *	0.526	0.583	0.605
	Cut-off	3.99	3.83	3.31	3.37	---	2.87	---	---	---	2.71	2.67	2.18	1.59
	SEN	0.85	0.45	0.65	0.51	---	0.52	---	---	---	0.69	0.63	0.93	0.90
	r	0.511 *	0.194 *	−0.511 *	0.143 *	−0.068	0.032	0.063	0.040	0.049	−0.074	0.001		0.253 *
TyG	AUC	0.679 *	0.908 *	0.581 *	0.503	0.684 *	0.779 *	0.520	0.507	0.431	0.621 *	0.500	0.472	0.665
	Cut-off	9.22	9.36	9.54	10.38	9.23	9.20	---	9.29	---	9.60	8.91	---	8.05
	SEN	0.73	0.90	0.41	0.13	0.53	0.66	---	0.49	---	0.40	0.67	---	0.83
	R	0.215 *	0.743 *	−0.085	0.043	0.208 *	0.377 *	0.005	0.052	0.064	−0.065	0.106*		0.347 *

CRI-I = TC/HDLC, CRI-II = LDLC/HDLC; AIP = Log (serum triglyceride/serum HDLC); AC = (TC-HDLC)/HDLC; TyG = Ln [fasting triglycerides (mg/dL) × fasting blood glucose (mg/dL)/2].

**Table 5:** Prevalence of cardiometabolic risk factors in mixed-hypercholesterolemic (HC: ≥200 mg/dL) populations: HC–Diabetic (A1c ≥ 5.7%), HC–HT (F. T4 < 12 pmol/L), HC–HD (Vit.D < 20 ng/mL) HC–HTN (BP ≥ 140/90), and HC–Anaemic (Hb < 12 in males, <13.5 in females), recording values higher than those of the cut-off compared to each respective population. NS means nonsignificant value of Chi^2^. Asterisk (*) denotes significant Chi^2^ at *p* < 0.05. † means risky level of the parameter.

(Cut-Off)	Cardiometabolic Risk Factors																			
	CRI-I (4.99)				CRI-II (2.47)				AC (3.99)				AIP (0.71)				TyG (9.22)			
Mixed-HC Population	No Risk		Risky		No Risk		Risky		No Risk		Risky		No Risk		Risky		No Risk		Risky	
	N	%	N	%	N	%	N	%	N	%	N	%	N	%	N	%	N	%	N	%
HC (Total)	46	47.4	51	52.6	42	46.7	48	53.3	44	45.8	52	54.2	52	52.0	48	48.0	43	46.7	49	53.3
HC/HT: † F. T4	33	47.8	36	52.2	30	44.1	38	55.9	32	47.1	36	52.9	36	52.2	33	47.8	29	46.0	34	54.0
HC/DM: † A1c	35	45.4	42	54.6	34	45.9	40	54.1	34	44.7	42	55.3	41	51.9	38	48.1	36	48.0	39	52.0
HC/HD: † Vit.D	25	53.2	22	46.8	21	48.8	22	51.2	23	50.0	23	50.0	28	57.1	21	42.9	27	57.4	20	42.6
HC/HTN: † BP	11	40.7	16	59.3	8	29.6	19	70.4	12	42.9	16	57.1	11	40.7	16	59.3	7	30.4	16	69.6
HC/anemic–Hb	14	60.9	9	39.1	8	40.0	12	60.0	14	60.9	9	39.1	16	64.0	9	36.0	14	60.9	9	39.1
Chi^2^, df, P	5.49, 1, (*p* = 0.019 *)				0.06, 1, (*p* = 0.803 ^NS^)				5.23, 1, (*p* = 0.022 *)				4.35, 1, (*p* = 0.039 *)				7.54, 1, (*p* = 0.006 *)			
HC/Males	12	36.4	21	63.6	16	51.6	15	48.4	10	31.2	22	68.8	13	39.4	20	60.6	14	43.7	18	56.3
HC/Females	29	64.4	16	35.6	19	47.5	21	52.5	29	64.4	16	35.6	34	70.8	14	29.2	25	61.0	16	39.0
HC/Age: <40 Y.	4	66.7	2	33.3	1	16.7	5	83.3	4	66.7	2	33.3	4	66.7	2	33.3	2	40.0	3	60.0
HC/Age: ≥40 Y.	9	36.0	16	64.0	9	36.0	16	64.0	9	36.0	16	64.0	9	36.0	16	64.0	6	30.0	14	70.0
HC/Obese: ≥30 kg/m^2^	2	33.3	4	66.7	2	33.3	4	66.7	2	33.3	4	66.7	2	33.3	4	66.7	0	0.0	6	100
HC/O.W: 25–29.9 kg/m^2^	3	21.4	11	78.6	5	35.7	9	64.3	3	21.4	11	78.6	3	21.4	11	78.6	4	28.6	10	71.4

## Data Availability

Not applicable.

## Abbreviations

Metabolic syndrome (MetS), cardiovascular diseases (CVD), type 2 diabetes mellitus (T2DM), hemoglobin-A1C, fasting serum glucose (FSG), total cholesterol (TC), triglycerides (TGC), high-density lipoprotein-cholesterol (HDLC), low-density lipoprotein-cholesterol (LDLC), vitamin-D (Vit.D), thyroid-stimulating hormone (TSH), free tetra-iodothyronine (F.T4), hemoglobin (Hb), creatinine (Cr), blood pressure (Bp: systole (S)/diastole (D)), Diabetes mellitus (DM), Hypercholesteremia (HC), hypothyroidism (HT), hypertension (HTN). Castelli’s risk factors; are CRI-I and CRI-II, atherogenic risk factors; atherogenic index in plasma (AIP), atherogenic coefficient (AC), and triglyceride-glucose index (TyG).
